# Dangerous Lunatics at Large

**Published:** 1890-08-30

**Authors:** 


### Passing Topics.

### DANGEROUS LUNATICS AT LARGE.

The application reported in the newspapers as having been made to a police magistrate by a practising physician in reference to a person suffering from homicidal mania illustrates a position of much gravity. In the case in point, the physician complained that, notwithstanding one of the parochial officers had been informed of the fact that a dangerous person was at large, he declined to take any steps in the matter, and when his attention was specifically drawn to his responsibility, he stated that "he regarded the message to him as an impertinent one."

To the parochial mind, things the most apposite often appear as impertinences, and we are not concerned with this particular example of a characteristic tendency to refuse evident but inconvenient conclusions; further than that it involves in the present connection a source of very considerable danger to the public.

Ever since the Weldon decision there has been a growing difficulty about obtaining the certificates necessary to the restraint of an alleged lunatic, and we are not backward to perceive that this is far from an unmixed evil. There is something startling in the thought when it is first brought home to us that it is possible for two men possessed of a medical qualification to inflict the taint of lunacy upon any person whom they, in their infallibility—for the power allowed them virtually establishes this—may adjudge to be of unsound mind.

But this consideration, which is wholly reasonable, and has exercised the imagination of not a few clever people, is out of place in regard to such circumstances as we have before us, and in which homicidal violence has occurred. A person who, while the subject of an attack of mania can be shown to have attempted his own life or someone else's is in an entirely different position from one who is the object of anxiety because of some harmless delusion or amiable eccentricity, even though its indulgence is troublesome, and it is evident there should be some speedy way of imposing the restraint needful to his own or other people's safety.

Yet, as the report shows, considerable difficulty lies in the way of prompt action, and it would seem as if there were no summary means of enforcing responsibility. In the case of a hospital the difficulty is greater than with a private individual. Instances have occurred wherein the only way of obtaining relief from a dangerous patient has been by the expedient of sending him home, and arming the friends with a strongly-worded intimation for them to lodge with the parish officer. Sometimes it happens that a patient, dangerous and able-bodied, will demand his discharge from the hospital, and rarely, if ever, will the parish move when applied to. So common is the trouble that it is the practice, at one hospital at least, to take no further step beyond calling the attention of the nearest police-constable to the patient as he goes away, a proceeding devised, we suppose, as a concession to conscience and the proprieties, but which, in the absence of a definite charge, has no practical value. In some instances, failing all means of riddance, persons of suicidal and violent tendencies, have remained in wards for sick persons during considerable periods, to the terror of other patients.

It is a remarkable habit of the average parish official to adopt an attitude of hostility towards a voluntary hospital. That a real danger is constituted by this indisposition to co-operate is obvious to the most casual observer, but apparently nothing short of a catastrophe will compel attention in official quarters. Notwithstanding the risk we run of being deemed guilty of an impertinence, we will ask whose is to be the responsibility, and how is that responsibility to be enforced when the relieving officer, the medical officer, or other representative of the parish, leaves a maniacal patient in unsuitable lodgings until he does violence to himself, or, may be, runs amuck among a ward of sick people?

Agricola.

